# Maternal depression in rural Pakistan: the protective associations with cultural postpartum practices

**DOI:** 10.1186/s12889-020-8176-0

**Published:** 2020-01-15

**Authors:** Katherine LeMasters, Nafeesa Andrabi, Lauren Zalla, Ashley Hagaman, Esther O. Chung, John A. Gallis, Elizabeth L. Turner, Sonia Bhalotra, Siham Sikander, Joanna Maselko

**Affiliations:** 1 Department of Epidemiology, Gillings School of Global Public Health, University of North Carolina at Chapel Hill, McGavran-Greenberg Hall, CB# 7435, Chapel Hill, North Carolina NC 27599 USA; 2 Carolina Population Center, University of North Carolina at Chapel Hill, 123 W. Franklin St, Chapel Hill, North Carolina NC 27516 USA; 3 Department of Sociology, University of North Carolina at Chapel Hill, 102 Emerson Dr, Chapel Hill, North Carolina NC 27514 USA; 4 School of Public Health, Yale University, 60 College St, New Haven, CT 06510 USA; 5 Department of Biostatistics and Bioinformatics, Duke University, 2424 Erwin Road, Durham, North Carolina NC 27705 USA; 6 Duke Global Health Institute, Duke University, Durham, North Carolina USA; 7 Department of Economics and Institute for Social and Economic Research, University of Essex, Wivenhoe Park, Colchester, CO4 3SQ UK; 8 Human Development Research Foundation, H 06, Street 55, Sector F-7/4, Islamabad, 44000 Pakistan; 9 Health Services Academy, Islamabad, Pakistan

**Keywords:** Postpartum, Perinatal, Depression, Mental health, Practices, Support, Pakistan

## Abstract

**Background:**

Traditional postpartum practices are intended to provide care to mothers, but there is mixed evidence concerning their impact on postpartum depression (PPD). It remains unknown if there is a unique impact of postpartum practices on PPD separately from other types of social support, or if practices differentially affect those with existing prenatal depression. In Pakistan, chilla (چله) is a traditional postpartum practice in which women receive relief from household work, additional familial support, and supplemental food for up to 40 days postpartum. This study aims to understand if chilla protects against PPD independent of other support and whether this relationship varies by prenatal depression status.

**Methods:**

Data come from the Bachpan cohort study in rural Pakistan. Chilla participation and social support (Multidimensional Scale of Perceived Social Support) were assessed at 3 months postpartum. Women were assessed for major depressive episodes (MDE) with the Structured Clinical Interview, DSM-IV and for depression symptom severity with the Patient Health Questionnaire (PHQ-9) in their third trimester and at 6 months postpartum. Adjusted linear mixed models were used to assess the relationship between chilla participation and PPD.

**Results:**

Eighty-nine percent of women (*N* = 786) participated in chilla and almost 70% of those that participated took part in all of chilla’s aspects. In adjusted models, chilla participation was inversely related to MDE (OR = 0.56;95%CI = 0.31,1.03) and symptom severity (Mean Difference (MD) = − 1.54;95%CI: − 2.94,-0.14). Chilla participation was associated with lower odds of MDE (OR = 0.44;95%CI = 0.20,0.97) among those not prenatally depressed and with lower symptom severity among those prenatally depressed (MD = -2.05;95%CI:-3.81,-0.49).

**Conclusions:**

Chilla is inversely associated with both MDE and symptom severity at 6 months postpartum above and beyond social support. Specifically, chilla is inversely associated with MDE among those not prenatally depressed and with lower symptom severity among those prenatally depressed. This relationship signals an opportunity for interventions aimed at preventing and treating PPD in this region to draw upon chilla and similar traditional postpartum practices in creating community-based, low-cost, sustainable interventions for maternal mental health.

**Trial registration:**

NCT02111915. Registered 18 September 2015. NCT02658994. Registered 22 January 2016. Both trials were prospectively registered.

## Background

The postpartum period is one of vulnerability for women’s mental health, which may be protected through pre-existing sociocultural practices. The importance of traditional postpartum practices to women’s postpartum experience has long been documented throughout regions of the world [1]. While such practices vary cross-culturally, the first months after giving birth are widely acknowledged as distinct from normal life and as a recuperative time [2]. Postpartum practices are richly documented in Asian, Hispanic, African, and Jewish cultures and are described by anthropologists and cultural psychiatrists as customs that “mother the mother” through formalized social support [1, 3–5]. They are intended to provide care to mothers by limiting their household activities, providing a special diet, taking care of their needs, and easing their transition into motherhood [1].

Such practices may also have long term benefits on maternal and infant health, including maternal mental health, and need to be considered when thinking about protections against postpartum depression (PPD) [1, 4, 6]. PPD is defined as depression beginning in or extending into the postpartum period, up to 1 year after giving birth, and can impair women’s functioning and the development of her children [7]. In low- and middle-income countries (LMIC), the overall pooled prevalence of PPD is between 18.7–19.8% [8, 9]. Though postpartum practices in Asian cultures are intended as supportive mechanisms for new mothers and the baby, there is mixed evidence concerning the impact of postpartum practices on PPD.

For instance, a systematic review on postpartum practices found that appropriate and wanted support may be protective against PPD. [10] In Taiwan, research suggests that women who engaged in traditional practices were less likely to exhibit physical symptoms and suffer from PPD at four to 6 weeks postpartum [11]. Yet, another systematic review of 16 studies reporting on traditional practices and PPD in Asian cultures found mixed results with eight studies reporting protective associations, four reporting negative associations, and the remaining studies were inconclusive [12]. Additional studies from Japan and Vietnam reported null associations and another from Singapore suggests that postpartum practices were correlated with higher depression levels [13–15].

It is likely that the potential effects of postpartum practices on PPD are closely tied to the social support components of such practices [10]. Social support refers to any type of assistance received from others, and is frequently categorized into instrumental and emotional support. There are known behavioral, psychological, and biological mechanisms through which both instrumental and emotional support act to influence health outcomes [16]. Postpartum practices provide instrumental support, such as a supplemented diet and relief from chores, and emotional support, such as a bolstered sense of self-worth. These social support components of postpartum practices may be driving the association between participation and reduced risk of PPD.

Prior literature has found that social support is often considered the most important aspect of participation in postpartum practices [10]. Participation may be less beneficial when support is less positive, such as when the practice creates conflict with a mother-in-law, or when support is unwanted. Yet, few studies that assess participation in postpartum practices have measured social support separately to tease out the complex relationships between engagement in postpartum practices and social support.

Additionally, prior work has focused on the role that postpartum practices play in preventing PPD onset, but has not assessed how these practices reduce pre-existing depression symptom severity [10, 12]. The pooled prevalence of women with PPD in LMIC is between 18.7–19.8% and the pooled prevalence of women diagnosed prenatally is between 15.6–19.2%, indicating that many women enter the postpartum period and begin their postpartum practices already experiencing depression [8, 9]. Thus, there is a need to understand if postpartum practices reduce depressive symptom severity among those prenatally depressed as well as prevent the onset of new symptoms.

In Pakistan, postpartum practices are referred to as chilla (چله) and are common after a women’s first birth [17]. Chilla is defined as a 40-day period of confinement after childbirth in which a woman returns to her mother’s home, is fed fortifying foods, is exempt from household responsibilities, stays indoors, and receives additional support. Yet, not all women return to their maternal home during this period; many resume chores within 7 days postpartum, and women may only receive unsolicited social support [18]. Additionally, PPD is estimated to be 30.9% in Pakistan, notably higher than the estimated prevalence of 18.7–19.8% in LMIC [19]. To date, one study in Pakistan has found chilla to be protective against PPD. [20] Given the dearth of evidence and inconclusiveness, our study of mothers in rural Pakistan aims to understand [1] how chilla affects depression diagnosis and depressive symptom severity, [2] whether this relationship exists above and beyond perceived social support and [3] whether this relationship varies by prenatal depression status.

## Methods

### Study design and participants

Data come from the Bachpan study, a cluster randomized perinatal depression trial nested in a longitudinal birth cohort in rural Pakistan. The study is representative of a typical low-socioeconomic rural area in the north of the Punjab Province. A more detailed description of the study is available elsewhere [21]. Briefly, pregnant women in 40 village clusters were identified and invited to be screened for depression using the Patient Health Questionnaire (PHQ-9). Women who scored 10 or higher were invited to participate in the study; roughly one in three women who scored less than 10 were asked to participate in the study [21]. Given unequal probabilities of selection into the study, sampling weights were used to represent the population of pregnant women in the area [21]. Women were then followed up at three, six, 12, 24, and 36 months postpartum through in-person interviews. We used data from pregnancy (baseline), three, and 6 months postpartum. Of the 1154 women interviewed at baseline, 331 were not interviewed at the three-month follow-up visit, resulting in 823 women in our analytic sample.

### Measures

#### Outcomes

Our main outcomes of interest were major depressive episode (MDE) and depressive symptom severity at 6 months. We utilized the Structured Clinical Interview for the Diagnostic and Statistical Manual of Mental Disorders’ (SCID-IV, hereafter referred to as SCID) Module for Current Major Depressive Episode to evaluate MDE [22]. The major depressive section of the SCID has been translated into Urdu, culturally adapted for Pakistan, and has been used cross-culturally with pregnant women [23, 24]. The SCID was collected at baseline, three, and 6 months. The PHQ-9 screens for depressive symptoms using nine items with a score ranging from zero to three for each item; individuals can receive a maximum score of 27. A 10-point cutoff is commonly used to indicate symptoms reaching a clinically significant level [25]. This measure has demonstrated acceptable criterion-related validity and reliability for screening depressive symptoms in our population of community-based Pakistani pregnant women [25]. The PHQ-9 was collected at baseline, three, and 6 months and treated as a continuous variable.

#### Exposure

At the 3 month postpartum interview, women were asked whether they participated in chilla. If yes, they were asked a series of questions about their chilla experience, which were based on a prior Pakistani study [26]. These included [1] the duration of their chilla (approximately one-week intervals from 1 to 40 days); three questions about chilla components: [2] being relieved from chores, [3] receiving additional female support, and [4] receiving a supplemented diet; and [5] a final question about whether the woman was satisfied with her chilla. Our primary analyses used the binary variable for chilla participation, as there was little variation in the component indicators [20]. Additional sensitivity analyses were conducted to assess whether specific components of chilla participation were associated with MDE at 6 months.

#### Covariates

The Multidimensional Scale of Perceived Social Support (MSPSS) is used globally and captures perceived support from family, friends, and significant others [27]. Informed by a prior validation study of the Urdu translation of the MSPSS among new mothers in rural Pakistan, we summed the scores of all 12 items to obtain a single summary measure of perceived social support [28]. The MSPSS was measured at 3 months postpartum.

We also adjusted for potential baseline confounders a priori: maternal age (years), maternal education (years), parity, socioeconomic status (SES) (a composite of household assets), and living with mother-in-law [29]. Our analysis also controlled for baseline outcome measures (e.g. SCID at baseline was included when SCID at 6 months was the outcome). In our models, we adjusted for study arm and the number of people living in a room, as this was associated with missingness at three- and six-month interviews.

#### Statistical analysis

We first present descriptive statistics for the sample characteristics and mean differences in these characteristics by chilla participation. Our primary analyses assessed the relationship between chilla participation and MDE (SCID) and depressive symptom severity (PHQ-9) at 6 months separately. We utilized multilevel mixed effects models with a logit link for SCID and an identity link for PHQ-9. All models used a random intercept to account for clustering at the village level. Given the unequal probabilities of selection into the study, sampling weights were used in models and descriptive statistics to represent the underlying population of pregnant women in the area. Our analyses were stratified by MDE at baseline, given that the relationship of chilla on six-month depression may be different among depressed and non-depressed mothers. We conducted two sensitivity analyses: [1] to examine the associations between specific components of chilla and MDE (SCID) and [2] to assess whether inclusion of MSPSS attenuated the association between chilla and subsequent depression. All analyses were conducted using Stata 15.

## Results

### Descriptive statistics

Our final analytic sample included 823 women who had complete data for baseline, three, and six-month timepoints. A comparison of baseline characteristics among women with and without available data is Table 4 in Appendix. After applying population-representative weights, women were, on average, 26.62 years old and over half of women (51.38%) had at least a secondary education (Table 1). Most had at least one other child (61.81%) and lived with their mother-in-law (68.56%). Mean social support (MSPSS) score at 3 months was 4.74 (SD: 1.15). Thirty-seven percent of women were diagnosed with MDE (SCID) at baseline and 23.06% were diagnosed at 6 months postpartum. Mean PHQ-9 score was 8.73 at baseline (SD: 6.68) and 4.70 at 6 months (SD: 5.44).

**Table 1:** Demographic, chilla, and depression characteristics, Bachpan cohort, Pakistan^a^

	Mean or N	SD or %	Min	Max
Demographic Characteristics (*N* = 823)				
Maternal Age	26.62	4.43	18	40
Maternal Education				
None	111	12.23		
Primary (1–5)	159	17.46		
Middle (6–8)	157	18.94		
Secondary (9, 10)	209	25.96		
Higher Secondary (11, 12)	84	10.92		
Tertiary (> 12)	103	14.50		
Parity				
First Pregnancy	238	30.60		
One or more children	585	69.40		
SES Quintiles				
Lowest	153	15.99		
Lower Middle	163	18.56		
Middle	171	21.13		
Upper Middle	165	21.64		
Highest	171	22.68		
Mother-in-law				
Yes	557	68.56		
People in Room	2.37	1.73	0	23
MSPSS 3 month	4.74	1.15	1	7
Chilla Characteristics (*N* = 729)				
Chilla				
Yes	729	89.44		
Duration				
1–7	71	9.06		
8–14	78	9.58		
15–20	120	15.79		
21–25	38	4.89		
26–30	53	7.01		
31–35	30	4.25		
36–40	339	49.42		
Chores				
Yes	620	85.30		
Female Support				
Yes	686	94.66		
Diet				
Yes	679	93.84		
Satisfaction				
Yes	602	84.79		
Depression Characteristics (*N* = 823)				
SCID (Baseline)	405	37.54		
SCID (Six months)	217	23.06		
PHQ-9 (Baseline)	8.73	6.68	0	27
PHQ-9 (6 months)	4.70	5.44	0	27

^a^ Percentages are based on weighted data to account for the sampling design in which 1 in every 3 non-depressed pregnant women were eligible for study inclusion; Abbreviations: Multidimensional Scale of Perceived Social Support (*MSPSS*), Socioeconomic Status (*SES*), Patient Health Questionnaire (*PHQ-9*), Structured Clinical Interview for DSM-IV (*SCID*)

### Chilla participation

The majority of women (89.44%) participated in chilla and over 80% were satisfied with their chilla experience (Table 1). Most participated for at least 26 days (60.68%) and almost 70% participated in all three components (relief from chores, additional female support, supplemented diet). Women who participated in chilla had, on average, more education, higher SES, were more likely to be in their first pregnancy, and more baseline social support. They also had fewer MDEs at baseline and lower depression symptom severity (Table 2). We also assessed whether baseline MDE (SCID) predicted chilla participation and chilla components. More women who were non-depressed at baseline participated in chilla, had longer chilla duration, and were more satisfied with their chilla experience (Table 5 in Appendix).

**Table 2:** Background Characteristics by Chilla Participation, Bachpan cohort, Pakistan*

	Chilla Participation		No Chilla Participation		Test of Difference	
	*N* = 729		*N* = 94		T-Test	Chi-Squared
	Mean or N	SD or %	Mean or N	SD or %		
Demographic Characteristics						
Maternal Age	26.68	4.38	26.19	4.79	−1.00	
Maternal Education						15.46**
None	96	13.17	15	15.96		
Primary (1–5)	130	17.83	29	30.85		
Middle (6–8)	136	18.66	21	22.34		
Secondary (9, 10)	195	26.75	14	14.89		
Higher Secondary (11, 12)	76	10.43	8	8.51		
Tertiary (> 12)	96	13.17	7	7.45		
Parity						4.93*
First Pregnancy	220	30.18	18	19.15		
One or more children	509	69.82	76	80.85		
SES Quintiles						17.61***
Lowest	125	17.15	28	29.79		
Lower Middle	138	18.93	25	26.60		
Middle	152	20.85	19	20.21		
Upper Middle	155	21.26	10	10.64		
Highest	159	21.81	12	12.77		
Mother-in-law						1.73
Yes	499	68.45	58	61.70		
People in Room	2.36	1.76	2.45	1.48	0.45	
MSPSS (Three months)	4.57	1.27	3.82	1.39	−5.31***	
Depression Characteristics						
SCID (Baseline)	350	48.01	55	58.51		3.67
PHQ-9 (Baseline)	8.56	6.62	10.05	6.96	2.04*	

*p < .05, ** < .01, ***p < .00; Abbreviations: Multidimensional Scale of Perceived Social Support (*MSPSS*), Socioeconomic Status (*SES*); Patient Health Questionnaire (*PHQ-9*), Structured Clinical Interview for DSM-IV (*SCID*)

### Symptom severity and MDE diagnosis

In unadjusted models, chilla participation was inversely related to both MDE (Table 3 - Panel A, Model 1: OR = 0.40; 95% CI = 0.23,0.71) and symptom severity (Table 3 - Panel B, Model 1: Mean Difference (MD) = − 2.51; 95% CI: − 4.20,-0.82). After adjusting for baseline covariates, we found that these relationships hold for both MDE (Table 3 - Panel A, Model 2: OR = 0.53; 95% CI = 0.33,0.86) and symptom severity (Table 3 - Panel B, Model 2: MD = -2.03; 95% CI: − 3.10,-0.96). We then controlled for social support and, although our estimate for chilla becomes slightly less precise, chilla remains independently related to MDE (Table 3 - Panel A, Model 3: OR = 0.56; 95% CI = 0.31,1.03) and symptom severity (Table 3 - Panel B, Model 3: MD = -1.54; 95% CI: − 2.94,-0.14). We conducted sensitivity analyses to disentangle potentially different associations of chilla components and PPD, adjusting for other components, and found no significant differences among the components (Table 6 in Appendix); however, this is likely because the majority of women who participated in chilla participated in all three components.

**Table 3:** Chilla Participation and Postpartum Depression at Six Months, Bachpan cohort, Pakistan†

	Model A: Full Sample - Unadjusted			Model B: Full Sample - MSPSS Excluded			Model C: Full Sample - Adjusted			Model D: Non-Depressed			Model E: Depressed		
	*N* = 823			*N* = 823			*N* = 823			*N* = 418			*N* = 405		
Panel A: SCID+															
	OR	CI		OR	CI		OR	CI		OR	CI		OR	CI	
Chilla (yes)	0.40	0.23	0.71	0.53*	0.33	0.86	0.56	0.31	1.03	0.44*	0.20	0.97	0.73	0.39	1.34
MSPSS							0.72***	0.61	0.85	0.66**	0.51	0.86	0.78*	0.65	0.93
SCID (Baseline)				2.47***	1.75	3.49	2.30***	1.62	3.26						
Maternal Age				1.01	0.97	1.06	1.02	0.98	1.07	0.98	0.92	1.05	1.02	0.97	1.07
Maternal Education				0.83**	0.72	0.94	0.81**	0.70	0.94	0.81*	0.66	1.00	0.88	0.74	1.04
Parity				1.20	0.78	1.84	1.11	0.64	1.92	1.37	0.71	2.64	1.22	0.68	2.18
SES				0.88	0.76	1.02	0.91	0.75	1.11	1.01	0.79	1.28	0.84	0.70	1.01
Mother-in-law				1.15	0.79	1.69	1.26	0.84	1.88	1.46	0.76	2.80	1.08	0.66	1.76
People in Room				1.00	0.91	1.10	1.01	0.91	1.12	1.09	0.96	1.23	0.92	0.80	1.06
Study Arm				0.85	0.59	1.23	0.89	0.60	1.33	0.94	0.54	1.61	0.85	0.56	1.29
Intercept	0.63	0.36	1.12	0.42	0.12	1.44	1.32	0.23	7.63	3.93	0.40	38.44	2.84	0.51	15.86
Panel B: PHQ-9++															
	Coefficient	CI		Coefficient	CI		Coefficient	CI		Coefficient	CI		Coefficient	CI	
Chilla (yes)	−2.51	−4.20	−0.82	−2.03***	−3.10	−0.96	−1.54*	−2.94	−0.14	−0.66	−1.98	0.67	−2.05*	−3.81	−0.49
MSPSS							−0.85***	−1.16	− 0.55	− 0.65**	− 1.06	− 0.24	− 0.93***	− 1.38	− 0.46
PHQ-9 (Baseline)				0.28***	0.22	0.33	0.25***	0.19	0.30	0.27***	0.17	0.36	0.21***	0.10	0.33
Maternal Age				0.04	−0.04	0.13	0.04	− 0.04	0.12	0.02	−0.08	0.12	0.04	−0.09	0.19
Maternal Education				−0.34	−0.60	− 0.07	−0.22	− 0.49	0.05	− 0.23	− 0.51	0.06	−0.37	− 0.81	0.07
Parity				0.09	−0.76	0.93	0.12	−0.55	0.78	0.21	−0.68	1.10	0.20	−1.34	1.00
SES				−0.11	−0.40	0.19	0.01	−0.36	0.37	0.11	−0.23	0.45	−0.12	−0.60	0.35
Mother-in-law				0.24	−0.55	1.02	0.26	−0.46	0.98	0.03	−0.85	0.91	0.36	−0.56	3.53
People in Room				0.04***	−0.16	0.25	0.05	−0.13	0.23	−0.05	−0.27	0.17	0.11	−0.22	0.48
sStudy Arm				−0.74	−1.44	−0.04	− 0.70	−1.37	−0.04	− 0.76	− 1.52	0.00	− 0.75	−1.88	0.31
Intercept	6.40	4.71	8.08	4.02*	1.45	6.59	7.34	4.24	10.44	6.13	2.67	9.58	8.98	4.52	14.00

† Models A-C used sampling weights and all models accounted for clustering due to the study design; *p < .05, ** < .01, ***p < .001; + Panel A used multilevel mixed-effects logistic regression with random effects to account for clustering; ++ Panel B used generalized linear mixed models with random effects to account for clustering; Abbreviations: Multidimensional Scale of Perceived Social Support (*MSPSS*), Socioeconomic Status (*SES*), Patient Health Questionnaire (*PHQ-9*), Structured Clinical Interview for DSM-IV (*SCID*)

To examine whether the association between chilla and depression differs by baseline MDE, we stratified models by baseline MDE diagnosis. In multivariable models that also included social support, we found that the association between chilla and MDE at 6 months was driven by those not clinically depressed at baseline (Table 3 - Panel A, Model D: OR = 0.44; 95% CI = 0.20,0.97) as opposed to those who were clinically depressed at baseline (Table 3 - Panel A, Model E: OR = 0.73; 95% CI = 0.39,1.34). Conversely, the association between chilla and symptom severity score was greater among those who had prenatal MDEs (Table 3 - Panel B, Model E: MD = -2.05; 95% CI: − 3.81,-0.49) compared to those who did not (Table 3 - Panel B, Model D: MD = -0.66; 95% CI: − 1.98,0.67).

## Discussion

### Postpartum practices’ differential effects

Among women in rural Pakistan, we found that most participated in chilla and that most participated in all components for the majority of the traditional 40 days. Those with more education, first-time mothers, and those with higher SES were more likely to participate in chilla, as well as those without MDEs and those with lower depression symptom severity at baseline. Among chilla participants, those without baseline MDEs had more positive chilla experiences.

We found that postpartum practices hold promise for benefiting maternal mental health. Chilla was inversely associated with depression at 6 months postpartum above and beyond social support. We saw a larger effect on symptom severity (PHQ-9) among those prenatally diagnosed with depression, indicating that postpartum practices may be particularly beneficial for women with already-vulnerable mental health. We also saw a negative association between chilla participation and SCID diagnosis at 6 months among those without MDEs at baseline, meaning that chilla participation may be protective against the future development of depression. However, we did not see an association among those with prenatal MDEs. Thus, chilla participation may help prevent new onset of PPD, but may not be enough to lift women out of pre-existing depression. Our findings may point to why prior research has been inconclusive in understanding the relationship between postpartum practices and maternal depression, as such practices may have differential effects depending on both the depression measure used (i.e., MDE or symptom severity) and women’s depressive history. Additionally, our findings may be specific to using the PHQ-9 and SCID. We used the PHQ-9 so that it would be generalizable across the prenatal, postnatal, and longer-term postpartum periods given the goal of the larger study. We chose to use the SCID-IV given its cross-cultural validation and use in this study setting [24, 30, 31]. Thus, studies using other instruments to assess symptom severity and MDE may generate different results.

### Chilla beyond social support

The negative association between participation in chilla and PPD in our study sample is consistent with prior findings in Pakistan, which found that the relative risk of depression among mothers who participated in chilla compared to those who did not participate was 0.4 (95% CI 0.3,0.6) [20]. Studies conducted in China, Vietnam, and Malaysia have also shown negative associations between participation in traditional postpartum practices and PPD. [14, 32, 33] Most often, social support is put forward as the mechanism linking traditional postpartum practices to reduced risk of PPD. For example, a study of mothers in Hong Kong found that the cultural practice of *peiyue* was associated with both better social support and lower risk of PPD. [32] After controlling for social support, *peiyue* was no longer associated with PPD, suggesting that social support is the “active ingredient” of *peiyue* [32]. However, in our study, we found that controlling for perceived social support did not appreciably attenuate the effect of chilla on depressive symptoms. While participation in chilla may be partially reflective of social support, we found that it was significantly associated with fewer depressive symptoms among women who were depressed during pregnancy regardless of their level of perceived social support. Thus, our results suggest that chilla captures more than perceived social support and may affect mental health through mechanisms other than social support.

It is likely that chilla affects mental health through multiple mechanisms, which are not fully captured by imagining chilla as a unidimensional proxy for social support. For example, mothers who are unable to participate in traditional postpartum practices may experience heightened role conflict, undermining their self-esteem [1]. Stern and Kruckman (1983) theorized that six primary elements influenced the presence or absence of PPD: a postpartum social support structure, recognition of vulnerability of the new mother, a mandated rest period, social seclusion, recognition of the role transition and social status of the new mother, and assistance with household tasks from female family, friends and midwives [1]. These specific cultural factors, they propose, buffer new mothers from PPD. In the Pakistani context, the postpartum practice of chilla encompasses these six primary protective elements, which suggests that we should consider the multiple dimensions of chilla and understand the full experience of chilla.

### The importance of postpartum practices

Our findings emphasize the importance of understanding informal postpartum practices for mothers that are central to the postpartum period in Pakistan and other countries [10]. Given how common these practices are in Asia and around the world, there is a specific need to better understand the role of postpartum practices on maternal mental health and well-being during this transition period for mothers and how we can incorporate these practices into mental health interventions [3–5]. There is also a need to identify the characteristics that predict mothers’ participation in these practices so that programs and interventions can focus on those less likely to participate. Supporting women’s participation in chilla aligns with a global interest in using pre-existing structures to provide additional social support to women experiencing depression in the pre and postpartum period [34, 35]. Enhancing the practice of traditional postpartum practices can offer a sustainable and culturally-appropriate public health recommendation, potentially in both high and lower income settings.

### Strengths, limitations, and future work

Our study has several strengths. First, it includes a longitudinal analysis from a population-representative sample. Second, we used standardized measures of depression symptom severity and MDE that have been validated in our target population. Third, it is the first, to date, to examine the effects of chilla participation, and one of the first to examine the effects of traditional postpartum practices, independently of social support on maternal mental health. Some limitations warrant discussion. First, as chilla and other postpartum practices are rarely quantitatively measured, it is not clear if our measurement of chilla captures the full experience. There is a need to more fully understand the quality and temporality of women’s chilla experiences. Second, due to the lack of variability among chilla components, we were unable to decipher what components may be driving the association we see with maternal depression besides social support (i.e., diet or length of participation). Third, we were unable to assess if there is something unique about women who did not participate in chilla, which was only 11%. Although our models control for social support and chilla components, there may be other elements of women’s social environment that we are unable to capture. We do see that women with less education, lower SES, and higher depression severity are less likely to participate in chilla. Thus, what presumably prohibited women from participating in chilla that we were unable to measure may be driving the association between chilla participation and PPD. Fourth, it is possible that child sex can affect whether a mother participates in chilla, as mothers of female children are less likely to receive social support in the absence of chilla [36]. However, our sample size was too small to separately assess female and male births. Future research should consider the potential for effect measure modification by child sex. Lastly, we may have selection bias in our analytic sample, as women absent at three-month data collection may have been participating in chilla. However, given that chilla typically does not last more than 40 days, this is unlikely at the three-month follow-up.

## Conclusion

Our study measures chilla’s relationship with diagnosed MDE and depression symptom severity. We find that chilla was inversely associated with depression at 6 months postpartum above and beyond social support. Chilla was inversely associated with MDE among those not prenatally depressed and with lower symptom severity among those prenatally depressed. It is important for public health programs aimed at reducing PPD to consider the importance of the postpartum practices already in place in many regions of the world. Our findings signal an opportunity for maternal mental health interventions to draw upon these longstanding practices in creating culturally appropriate interventions for maternal mental health.

## Data Availability

The datasets generated and/or analyzed during the current study are not publicly available due to ongoing data collection. However, data will be released upon completion of the study and, additionally, are available from the senior author Maselko on reasonable request.

## Appendix

**Table 4:** Comparison of baseline characteristics between analytic and missing samples, Bachpan cohort, Pakistan

	Analytic Sample		Reappear at 6 Months		Test of Difference+		Only in Baseline		Test of Difference++	
	*N* = 823		*N* = 105				*N* = 226			
	N/Mean	%/SD			T-Test	Chi-Squared	N/Mean	%/SD	T-Test	Chi-Squared
Demographic Characteristics										
Maternal Age	26.62	4.43	26.63	4.64	−0.01		27.05	4.91	−1.25	
Maternal Education						4.29				
None	111	12.84	18	17.14			41	18.14		3.47
Primary (1–5)	159	18.33	23	21.9			44	19.47		
Middle (6–8)	157	18.67	19	18.1			39	17.26		
Secondary (9, 10)	209	25.66	28	26.67			56	24.78		
Higher Secondary (11, 12)	84	10.35	5	4.76			20	8.85		
Tertiary (> 12)	103	14.16	12	11.43			26	11.50		
Parity						0.11				3.06
First Pregnancy	238	28.92	32	30.48			79.00	34.96		
One or more children	585	71.08	73	69.52			147.00	65.04		
SES Quintiles						4.67				4.07
Lowest	153	16.93	27	25.71			50	22.12		
Lower Middle	163	18.08	17	16.19			51	22.57		
Middle	171	20.66	16	15.24			44	19.47		
Upper Middle	165	21.51	21	20			45	19.91		
Highest	171	22.82	24	22.86			36	15.93		
Mother-in-law						0.33				4.45*
Yes	557	68.5	74	70.48			136	60.18		
People in Room	2.37	1.73	2.16	1.13	1.20		2.72	2.56	−2.36*	
Depression Characteristics										
SCID Baseline	405	49.21	52	49.52		0.00	105	46.88		0.38
PHQ-9 Baseline	8.73	6.68	8.22	6.79	0.74		8.65	6.82	0.16	

**p* < .05, ** < .01, ****p* < .001; + Test of difference between analytic sample and those reappearing at 6 months; ++ Test of difference between analytic sample and those only at baseline; Abbreviations: Socioeconomic Status (*SES*), Patient Health Questionnaire (*PHQ-9*), Structured Clinical Interview for DSM-IV (*SCID*)

**Table 5:** Comparison of chilla characteristics by baseline clinical depression, Bachpan cohort, Pakistan

	Non-Depressed Baseline		Depressed Baseline		Test of Difference	
	N/Mean	%/SD	N/Mean	%/SD	T-Test	Chi-Squared
	*N* = 418		*N* = 405			
Chilla						
Yes	379	90.67	350	86.42		3.67
	*N* = 379		*N* = 350			
Duration					3.79***	
1–7	28	7.39	43	12.29		
8–14	37	9.76	41	11.71		
15–20	52	13.72	68	19.43		
21–25	20	5.28	18	5.14		
26–30	27	7.12	26	7.43		
31–35	14	3.69	16	4.57		
36–40	201	53.03	138	39.43		
Chores						0.02
Yes	323	85.22	297	84.86		
Female Support						3.09
Yes	358	94.46	328	93.71		
Diet						0.18
Yes	359	94.72	320	91.43		
Satisfaction						16.89***
Yes	334	88.13	268	76.57		

**p* < .05, ** < .01, ****p* < .001

**Table 6:** Chilla participation and Postpartum Depression at Six Months, Bachpan cohort, Pakistan

	Model A: Full Sample - Unadjusted			Model B: Full Sample - Adjusted			Model C: Non-depressed			Model D: Depressed		
	*N* = 729			*N* = 729			*N* = 379			*N* = 350		
Panel A: SCID+	OR	CI		OR	CI		OR	CI		OR	CI	
Chilla - Duration	0.87***	0.80	0.94	0.92	0.84	1.01	0.87	0.75	1.01	0.95	0.85	1.06
Chilla - No Chores	0.69	0.39	1.23	0.62	0.36	1.04	1.02	0.43	2.42	0.53*	0.28	0.99
Chilla - Female Help	1.23	0.56	2.71									
Chilla - Diet	0.72	0.32	1.62	0.93	0.44	1.98	1.09	0.29	4.07	0.72	0.31	1.67
Chilla Satifaction	0.77	0.49	1.21	1.09	0.64	1.87	0.63	0.26	1.55	1.32	0.70	2.51
MSPSS				0.74**	0.60	0.91	0.72*	0.53	0.97	0.77**	0.63	0.95
SCID Baseline				2.42***	1.61	3.62	1.02	0.94	1.11	1.03	0.97	1.09
Maternal Age				1.04	0.99	1.09						
Maternal Education				0.84*	0.73	0.97	0.88	0.69	1.11	0.93	0.77	1.12
Parity				1.12	0.61	2.06	1.29	0.61	2.71	1.27	0.66	2.44
SES				0.90	0.73	1.10	0.95	0.72	1.25	0.83	0.68	1.02
Mother-in-law				1.35	0.81	2.25	1.68	0.80	3.55	1.10	0.64	1.89
People in Room				1.02	0.93	1.11	1.12	0.98	1.28	0.93	0.79	1.08
Study Arm				0.92	0.61	1.39	0.91	0.48	1.73	0.87	0.55	1.40
Intercept	0.98	0.38	2.49	0.82	0.09	7.70	0.92	0.05	17.88	3.08	0.35	27.02
Panel B: PHQ-9++	Coefficient	CI		Coefficient	CI		Coefficient	CI		Coefficient	CI	
Chilla - Duration	−0.22	− 0.45	0.01	− 0.04	− 0.27	0.18	− 0.02	− 0.22	0.18	− 0.06	− 0.34	0.21
Chilla - No Chores	− 0.56	−2.29	1.18	− 0.59	−2.05	0.87	0.47	− 0.69	1.64	−1.95*	−3.57	− 0.34
Chilla - Female Help	1.52	− 0.46	3.49									
Chilla - Diet	−2.48**	−4.40	−0.56	− 1.49**	− 2.63	− 0.34	− 1.97*	−3.75	− 0.19	−0.71	− 2.88	1.46
Chilla Satifaction	−0.61	− 2.02	0.79	0.13	− 1.30	1.55	−0.06	− 1.42	1.31	0.25	− 1.32	1.83
MSPSS				−0.69***	−1.01	−0.37	− 0.54**	−0.97	− 0.12	−0.80***	−1.31	− 0.29
PHQ-9 Baseline				0.24***	0.18	0.30	0.28***	0.19	0.38	0.20***	0.08	0.32
Maternal Age				0.05	−0.03	0.13	0.05	− 0.05	0.15	0.06	−0.08	0.20
Maternal Education				−0.17	−0.42	0.08	−0.20	− 0.50	0.10	− 0.30	−0.75	0.16
Parity				0.12	−0.54	0.78	0.19	−0.71	1.09	0.10	−1.45	1.64
SES				0.03	−0.33	0.38	0.12	−0.24	0.47	−0.06	−0.55	0.44
Mother-in-law				0.35	−0.32	1.01	0.32	−0.61	1.24	0.38	−0.95	1.71
People in Room				0.07	−0.10	0.24	−0.02	− 0.23	0.20	0.19	−0.17	0.54
Study Arm				−0.78*	−1.40	−0.15	− 0.91*	−1.70	−0.13	− 0.76	− 1.92	0.39
Intercept	6.88	4.85	8.91	6.65	3.40	9.89	5.40	1.34	9.46	7.91	2.27	13.54

**p* < .05, ** < .01, ****p* < .001; Abbreviations: Multidimensional Scale of Perceived Social Support (*MSPSS*), Socioeconomic Status (*SES*), Patient Health Questionnaire (*PHQ-9*), Structured Clinical Interview for DSM-IV (*SCID*)

## Abbreviations

LMIC: Low- and middle-income countries
MDE: Major Depressive Episodes**;**
MSPSS: Multidimensional Scale of Perceived Social Support
PHQ-9: Patient Health Questionnaire
PPD: Postpartum Depression
SCID: Structured Clinical Interview for the Diagnostic and Statistical Manual of Mental Disorders
SES: Socioeconomic Status
